# Laparoscopic subtotal cholecystectomy for difficult cases of acute cholecystitis: a simple technique using barbed sutures

**DOI:** 10.1186/s40792-020-01026-1

**Published:** 2020-09-29

**Authors:** Sho Fujiwara, Kenji Kaino, Kazuki Iseya, Nozomi Koyamada

**Affiliations:** Department of Surgery, Iwate Prefectural Chubu Hospital, 17-10 Murasakino, Kitakami, Iwate 024-8507 Japan

**Keywords:** Laparoscopic subtotal cholecystectomy, Barbed sutures, Acute cholecystitis, Tokyo Guidelines 2018

## Abstract

**Background:**

Laparoscopic cholecystectomy (LC) for difficult acute cholecystitis (AC) cases bears a high risk of vasculobiliary injuries (VBI). The Tokyo Guidelines 2018 (TG18) recommend the use of bailout procedures and subtotal cholecystectomy to prevent VBI. Performing a safe LC is challenging, even when followed by an accurate pre-surgical assessment. Laparoscopic cholecystectomy (LSC) requires advanced skills, and there is a risk of recurrence of cancer and/or gallbladder stones (GBS) in the remnant gallbladder (GB). Moreover, it is sometimes impossible to safely close the cystic duct with either a loop tie or linear staples because of anatomical and fragility problems. Here, we report a novel technique employing barbed sutures for LSC in difficult AC cases.

**Case presentation:**

We performed urgent LSC using barbed sutures for the stump of the cystic duct in two patients. In preoperative assessments, we found that these cases were qualified for operations rather than GB drainages, but the cystic ducts appeared difficult to close due to their severe inflammation and fragility during the operations. We applied barbed suture as a surrogate technique to close the stump of cystic duct. In patient 1, a 67-year-old woman with severe heart failure and type 2 diabetes mellitus was diagnosed with grade III AC. Pathological diagnosis was gangrenous cholecystitis. In patient 2, a 68-year-old woman who was referred to our hospital after 15 days of treatment for AC with antibiotics without drainage. The severity of AC was grade II according to TG18. Pathological diagnosis was acute-on-chronic cholecystitis. Both patients were discharged without complication.

**Conclusions:**

The utilization of barbed sutures in LSC stems as a feasible and safe surrogate technique. Furthermore, this approach could decrease the risks associated with the remnant GB.

## Background

Laparoscopic cholecystectomy (LC) for complicated acute cholecystitis (AC) cases is associated with a risk of vasculobiliary injuries (VBI) and requires advanced surgical skills. Even if LC is expected to be more useful than gallbladder (GB) drainage, performing it safely is rather challenging. The Tokyo Guidelines 2018 (TG18) indicate a clear strategy for such cases and recommend the use of bailout procedures and subtotal cholecystectomy as an optional approach to prevent VBI [1]. Although subtotal cholecystectomy is an important treatment option for difficult LC cases, it bears a risk of cancer or gallbladder (GB) stone recurrence in the remnant GB [2, 3]. Furthermore, in these cases, it is sometimes difficult to close the fragile cystic duct if remnant GB makes a minimum. Here, we report a novel technique employing barbed sutures for LSC in complicated AC cases.

## Case presentation

Since January 2020, we have performed urgent LSC using barbed sutures for the stump of the cystic duct according to TG18 in two patients. In our institute, our first choice is early LC for grade I, II, and III AC, if there is no contraindication; we perform urgent GB drainage for the AC with poor performance status or contraindication. Indication of the barbed suture technique is as follows: clips, endoloops, and linear staplers are unavailable because of the condition of cystic duct (thick or fragile) or anatomical difficulty (no secure a seam allowance width of the cystic duct or space). Contraindication of this technique is as follows: suture line cannot keep above the line between the base of Segment 4 and the roof of Rouviere’s sulcus.

Patient 1 was a 67-year-old woman with severe heart failure and type 2 diabetes mellitus. She was diagnosed with grade III AC, and the pathological diagnosis was gangrenous cholecystitis. Patient 2 was a 68-year-old woman who was referred to our hospital after 15 days of treatment for AC with antibiotics (piperacillin–tazobactam) without drainage. The severity of AC in this patient was grade II according to TG18, and the pathological diagnosis was acute-on-chronic cholecystitis. The clinical characteristics and perioperative data of the aforementioned patients are summarized in Table 1. Although we found that these cases were qualified for operations rather than GB drainages in preoperative assessments, the cystic ducts appeared difficult to close due to their severe inflammation and fragility.

**Table 1:** Clinical characteristics and perioperative data

	Patient 1	Patient 2
Sex	Female	Female
Age	67	68
Body mass index (kg/m^2^)	32.6	32.5
ASA score	4E	3E
TG grade	III	II
Preoperative severity assessment	Cardiovascular dysfunction (requiring treatment with dopamine and norepinephrine), hepatic dysfunction (PT-INR 1.68), CT finding (marked local inflammation; gangrenous cholecystitis and pericholecystic abscess)	Palpable tender mass in the right upper abdominal quadrant, duration of complaints; 15 days, CT finding (marked local inflammation; pericholecystic abscess)
Preoperative risk factors	Dilated cardiomyopathy (ejection fraction 27%), type 2 diabetes mellitus (HbA1c 9.2%), atrial fibrillation (rivaroxaban)	Hyperlipidemia, chronic heart failure
Operative time (min)	120	147
Estimated blood loss (ml)	10	5
Postoperative hospital stay (days)	12	5
Postoperative morbidity	None	None
Pathological diagnosis	Gangrenous cholecystitis	Acute-on-chronic cholecystitis

**Fig. 1:**
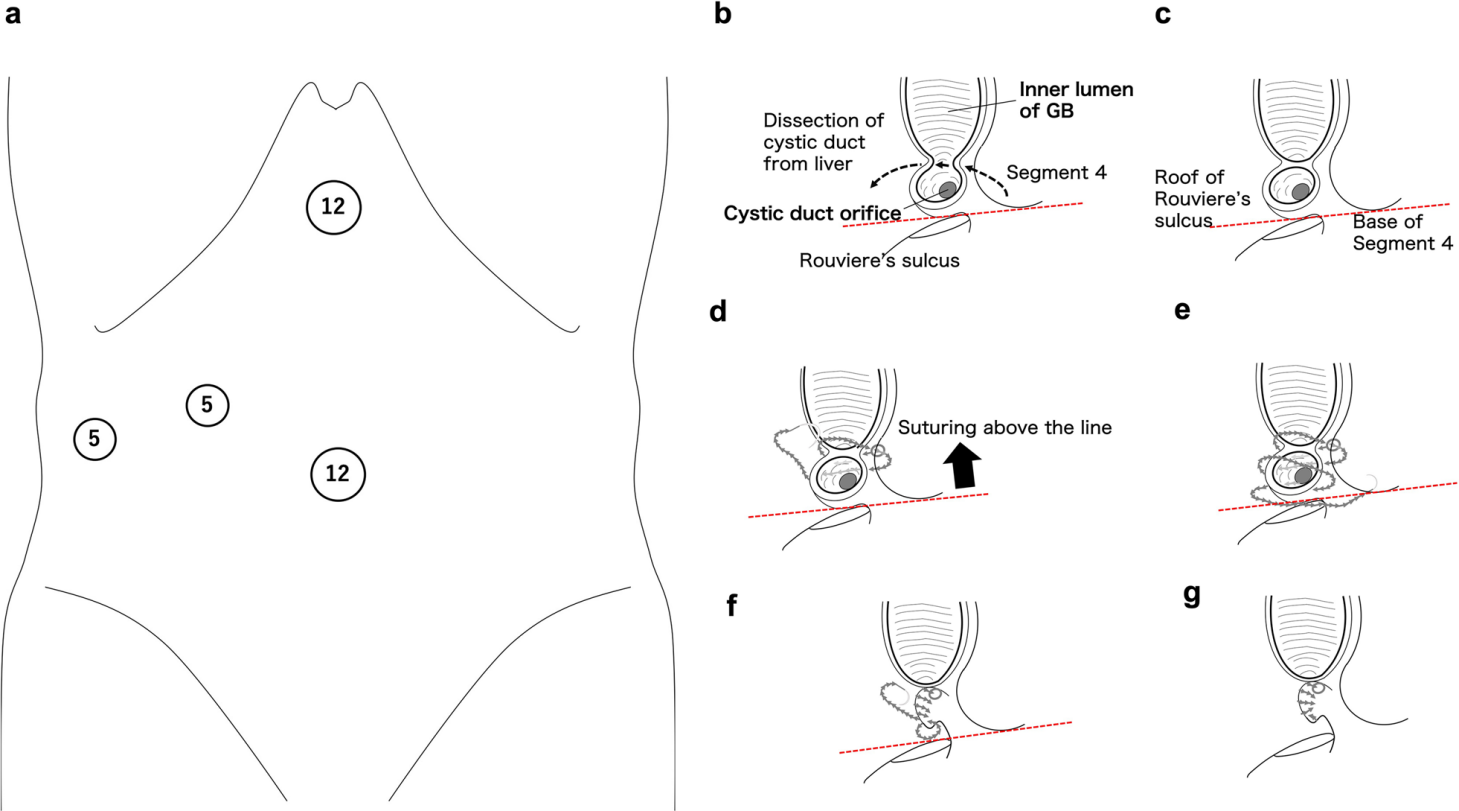
Port placement for LSC and surgical procedures of LSC. **a** The scopist used the umbilical port and main surgeon stood left side of patient. **b** Making incision on the GB and identification of cystic duct orifice from the inner lumen of GB leaving a part of GB wall on the liver bed. Dissection of cystic duct from the liver. **c** Isolation of cystic duct and identification of the line between the base of Segment 4 and the roof of Rouviere’s sulcus. **d**–**g** Suture using an absorbable 3-0 V-Loc above the line

**Fig. 2:**
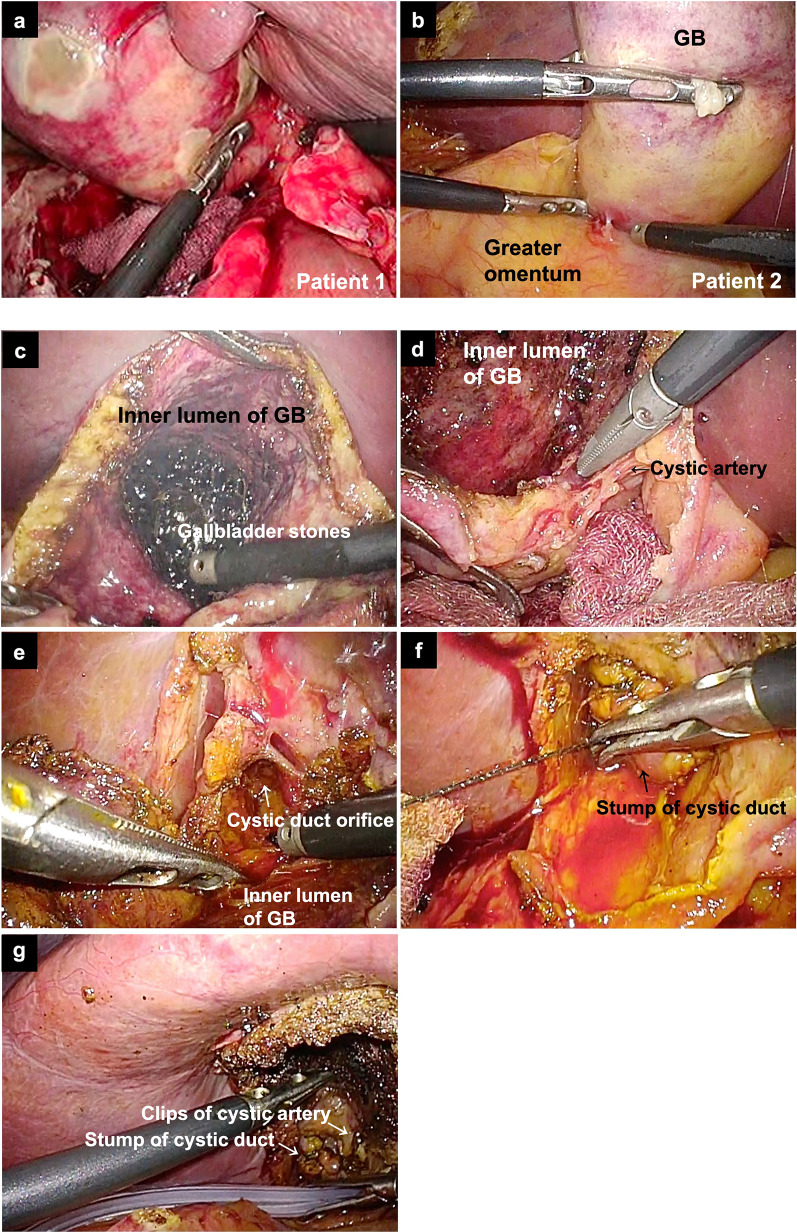
Intraoperative findings of Calot’s triangle and surgical procedures of LSC. **a** Calot’s triangle of patient 1. **b** Calot’s triangle of patient 2, and dissection of adhesion around GB and liver. **c** Making incision on the GB wall and aspiration of the contents in GB. **d** Identification of cystic artery and clipping. **e** Identification of cystic duct orifice from the inner lumen of GB after removal of GBS. **f** Suture using an absorbable 3–0 V-Loc. **g** Placement of drainage tube beside the stump.

The port placement employed in this study and procedures are shown in Fig. 1. The surgical procedures were based on bailout procedures for difficult AC cases stated in TG18. Both patients had a high risk of VBI and we could not perform the critical view of safety: severe inflammation and fibrosis in patient 1 (Fig. 2a), and severe fibrosis and scarring in patient 2 (Fig. 2b). We show the intraoperative findings in patient 2. We made an incision in the GB wall (Fig. 2c) and identified the cystic duct orifice from the inner lumen of the GB after removal of GBS and its contents (Fig. 2d, e). Then, we resected as much of the GB as possible to secure a seam allowance width of the cystic duct and minimum part of the GB neck. We resected the GB wall, considering the suture line, and left a minimum remnant of the GB wall of the neck near the cystic duct to be sutured using an absorbable 3–0 barbed suture, V-Loc™ (Covidien, Medtronic, Mansfield, MA, USA), and did not leave space for the stump (Fig. 2f). We placed a 6.5-mm round-type closed drainage tube next to the stump (Fig. 2g).

## Discussion

LC is a standard and well-established procedure; however, its application in difficult cases, such as severe AC, is controversial [4]. TG18 shows and specifies clear indications for employing LC depending on the severity of cases [1, 5]. For safety, bailout procedures are recommended by TG18 for performing LC; subtotal cholecystectomy is also an important treatment option, which avoids VBI. However, detailed guidelines for LSC have not been reported, and well-advanced skills of the surgeon are required.

Previous reports have shown various LSC procedures for GB stumps, including the use of large clips, endoloops, linear staplers, laparoscopic suturing of remnant GB, and simple drainage without closing the stump [2]. The thickened and fragile GB walls due to severe inflammation and/or fibrosis often make it difficult to deal with the stump of the remnant GB. In such cases, surgeons must choose either laparoscopic suturing, open conversion, or simple drainage [4]. Previous systematic reviews and meta-analyses have shown that LSC has a higher risk of bile leakage than open conversion, but perioperative complications including bile duct injury and mortality rate are low compared to the open conversion approach [2, 3]. Laparoscopic suturing may decrease the risk of bile leakage. Laparoscopic suturing is a feasible option for GB stumps even when the GB wall is thickened and fragile because of inflammation and when other devices cannot be utilized. However, it requires advanced skills of highly experienced surgeons. To the best of our knowledge, our technique of performing LSC using barbed sutures has not been reported before in PubMed’s databases. Furthermore, this technique is simple and enables an easy endoscopic suture of the cystic duct with minimum remnant GB. Only one previous Japanese case report presented LSC using barbed suture for chronic cholecystitis [6]. They could dissect GB from liver bed and encircle the cystic duct using GB body as a handle. However, our cases were difficult and severer AC cases; we could not even dissect GB from liver bed. In comparison to their procedure, our procedure can be applicable to the difficult AC cases and versatile procedure.

This technique is based on laparoscopic suturing, anastomosis, and reconstructions using barbed sutures in various fields of surgery. Some studies have reported the utilization of barbed sutures in repairing bile duct injuries and laparoscopic common bile duct exploration [7, 8]. Previous report suggested the barbed suture technique is well established and offers significant benefits in laparoscopic suturing, such as shortening of the operative and suturing durations [9, 10]. Systematic reviews and meta-analyses have previously shown this technique to be safe and beneficial, without increasing the incidence of complications related to suturing [11]. However, we have to consider some trouble due to barbed sutures; it may be extremely difficult to repair it if local damage or erroneous operation around biliary tract or vessels occurs. To prevent these troubles, suture line should be above the line between the base of Segment 4 and the roof of Rouviere’s sulcus [1]. Furthermore, we should be careful not to be bite size too much during suture. These principles minimize the incidence of troubles. These data may support the effectiveness and safety of the technique used in this study for difficult AC cases.

Our study has some limitations. First, this report does not discuss long-term outcomes of the LSC. LSC has been associated with the risk of GB cancer and GBS recurrence; however, the probability of these risks is low [2, 3]. In this study, we resected almost the entire GB, and the cystic duct with minimum remnant of the GB neck was sutured in order not to leave space for the stump, which differed from conventional reconstituting LSC procedures. The features of remnant GB in our study were similar to those in fenestrating LSC [12]. This may contribute to the decreased risk of GB cancer and GBS recurrence. Second, even though this technique enables easy suturing, we did not evaluate the suturing time compared with that of conventional suturing techniques. The procedures for the two cases in this study were performed by a sixth-year postgraduate fellow, and there were no technical interruptions during the procedures. Moreover, reports have already demonstrated the effectiveness of the barbed suture technique in shortening the suturing time in various fields of surgery [9, 10, 13, 14].

## Conclusion

This report describes a simple LSC technique in AC using barbed sutures for the stump of the cystic duct based on standard laparoscopic suturing techniques. The utilization of this technique may be feasible for complicated AC cases. Furthermore, this technique simplifies the suturing procedure without causing postoperative complications. Even if surgeons decide that LC is possible without GB drainages, cases with difficulty in closing the cystic duct stump are inevitable.

The utilization of barbed sutures in LSC for difficult AC cases is a technically feasible option. However, we must evaluate the long-term outcomes of this technique and investigate the perioperative data in more detail.

## Data Availability

The data that support the findings of this study are available from the corresponding author, SF, upon reasonable request.

## Abbreviations

AC: Acute cholecystitis
GB: Gallbladder
GBS: Gallbladder stones
LC: Laparoscopic cholecystectomy
LSC: Laparoscopic cholecystectomy
TG18: The Tokyo Guidelines 2018
VBI: Vasculobiliary injury
